# Feasibility of a brief mindfulness-based program for burnout in pain healthcare professionals

**DOI:** 10.3389/fpsyg.2022.1009266

**Published:** 2022-11-07

**Authors:** Anna Server, Carlos Suso-Ribera, Marcos Pérez-Carrasco, Javier Medel, Ángela Mesas, Alfonso Ayora, Rosa Maria Gracia

**Affiliations:** ^1^Department of Anesthesiology, Hospital Universitari Vall d'Hebron, Barcelona, Spain; ^2^Vall d’Hebron Research Institute, Universitat Autònoma de Barcelona, Barcelona, Spain; ^3^Department of Personality, Assessment and Psychological Treatments, Universitat Jaume I, Castelló, Spain; ^4^Institute of Health Carlos III, CIBERObn CB06 03/0052, Madrid, Spain; ^5^Department of Critical Care, Hospital Universitari Vall d'Hebron, Barcelona, Spain; ^6^Department of Occupational Health, Hospital Universitari Vall d’Hebron, Barcelona, Spain

**Keywords:** mindfulness, burnout, stress, health professionals, pain clinic

## Abstract

**Introduction:**

Stress inherent to health care, which is characterized by work overload and shortage of specialized staff, is associated with decreased quality of life and suboptimal patient care. Mindfulness-based programs have proved to be effective in reducing stress in healthcare providers. This study aims to assess the feasibility of an 8-week mindfulness program to reduce the burnout levels of the staff of a pain clinic in a tertiary public hospital.

**Materials and methods:**

A longitudinal study with a within subject pre/post-intervention design, consisting of daily face-to-face 10-min sessions and the creation of a virtual group using a social media platform. Variables measured: burnout, mindfulness, empathy, self-compassion, and demographic characteristics.

**Results:**

Program feasibility (i.e., reach, adherence, acceptability, and preliminary effectiveness) was evaluated in 10 participants (6 physicians, 2 nurse practitioners, 1 nursing assistant, and 1 administrative). The results revealed a high reach (i.e., participation rate of 90%), excellent adherence to the program (daily practice 95% of times), and very good acceptability of the group format and satisfaction with most treatment components. Regarding potential effectiveness, we report the results of the Wilcoxon signed-rank tests and its associated effect size (*r*). We observed improvements in mindfulness and all its subscales (−2.077 ≤ *Z* ≤ −2.703, 0.69 ≤ *r* ≤ 0.90, all *p* < 0.05) except for non-reactivity and all subcomponents of self-compassion (−2.501 ≤ *Z* ≤ −2.611, 0.83 ≤ *r* ≤ 0.87, all *p* < 0.05) but not on its global self-compassion score. Empathy and burnout did not change. In an exploratory manner, however, we found significant reductions in the burnout component of emotional exhaustion, but only in physicians (*Z* = −2.201, *p* = 0.028, *r* = 0.73).

**Discussion:**

We believe that the 8-week mindfulness-based program described in the present investigation might be a feasible and potentially effective method that can be easily implemented to reduce burnout and promote mindfulness in specialized pain clinics.

## Introduction

It is widely known that the stress inherent in health care is associated with unfavorable health and work outcomes in the healthcare professional, including lowered mood and decreased quality of life, unwillingness to work, job dissatisfaction, and suboptimal patient care practices (Suñer-Soler et al., 2014; Kumar, 2016; Salyers et al., 2017). In an attempt to summarize all these variables that can be affected by occupational stress in a single construct, the literature has converged towards the concept of burnout (Paris and Hoge, 2010).

Burnout is a complex syndrome characterized by depersonalization, emotional exhaustion, and a sense of low personal accomplishment (Maslach, 1993). Burnout impacts negatively both on the healthcare professional, as well as on the quality of patient care (Paris and Hoge, 2010). Burnout increases the risk of sick-leave absences, future systemic diseases, and mental and behavioural disorders. Consequently, calls have been repeatedly made to search for initiatives to enhance the healthcare professional’s well-being and to reduce this syndrome in this population (Toppinen-Tanner et al., 2005; Moss et al., 2016).

Burnout appears to be a very frequent syndrome in medical disciplines, including family physicians and surgeons, among others (Kumar, 2016). Only recently, there has been a growing awareness about the problem of occupational stress and burnout among anesthesiologists worldwide and research has highlighted the risk of different manifestations and consequences of burnout, which range from medical errors to suicide (De Hert, 2018).

As a result of the above, several initiatives have been developed to reduce burnout in healthcare professionals, including structural interventions (e.g., shortened attending rotation length and various modifications to clinical work processes) and individual-focused interventions (e.g., stress management and self-care training or communication skills training), with encouraging results (Luberto et al., 2017). Among the wide range of strategies available, brief mindfulness-based programs have gained ground in the past years due to their relatively reduced costs, moderate efficacy, healthcare acceptability, and their ability to enhance job performance (Gracia Gozalo et al., 2018; Pérez-Aranda et al., 2019; Xie et al., 2021).

The term mindfulness corresponds to the Pali term “sati,” which is a significant element of Buddhist traditions. In its original context, mindfulness is the ability to systematically understand one’s moment-to-moment experience and to develop self-knowledge through practice (Karunamuni and Weerasekera, 2017). Meditation techniques have been found of great value by Buddhists, Hindus, Muslims, or Christians for thousands of years and it has gained wide acceptance amongst non-religious public over the past few years. However, it should be mentioned that the contemporary understanding of mindfulness has been substantially simplified and divorced from its origins to fit current society requirements. In such contemporary understandings, mindfulness is seen as a state of mind in which one is highly aware of the present moment, observing what happens in a moment-to-moment manner, including one’s inner experiences, without evaluating or judging one’s experiences, and being highly compassionate with oneself (Bishop et al., 2004). The fact that this skill can be successfully trained improving inner peace and coupled with modern psychology approaches has enabled the development of specific stress management programs, such as Mindfulness-Based Stress Reduction (MBSR), originally developed by Kabat-Zinn (1982).

So far, the effectiveness of MBSR to reduce stress has been proven in different populations, including patients with emotional disorders (López-del-Hoyo et al., 2022) or chronic pain (Sanabria-Mazo et al., 2020), as well as in physicians from several specialties, psychologists, and nurses, among others (Franco et al., 2010; Aranda Auserón et al., 2017; Gracia Gozalo et al., 2018; Villarreal et al., 2021). Encouragingly, recent systematic reviews suggests that this intervention has the potential to significantly reduce stress in healthcare professionals (Burton et al., 2017; Lomas et al., 2019; Fendel et al., 2021). In particular, MBSR has been argued to be a program of choice for the reduction of burnout in healthcare professionals because in taps into key mechanisms involved in daily practice, such as attentional processes, decision making, and emotional competencies (Braun et al., 2019; Hanson et al., 2020; Weisbaum and Chadi, 2022). The goal of the present investigation is to explore whether these promising findings can be generalized to healthcare professionals attending an arguably different population, namely patients with chronic pain. Compared to other hospital units, pain clinics were more recently created and owe their existence to a conceptual change in the understanding of pain as a clinical entity, as opposed to only accompanying different pathologies (Bonica, 1953). Because of this reconceptualization of pain, specialized, multidisciplinary pain units have been created, where anesthesiologists collaborate with other healthcare specialists (e.g., nurses, psychologists, psychotherapists, and occupational therapists, among others) for a comprehensive management of pain.

Like other countries, pain clinics in Spain are often heterogeneous with respect to structure and equipment, including a variable number of staff members across units and different levels of multidisciplinarity, but they are often characterized by a majority of anesthesiologists. Work overload is frequent, as well as shortage of specialized pain staff, although this has not been properly documented. In addition to these potentially stressful factors, the personality and behavioral characteristics of persons with chronic pain, as well as the unrealistic expectations that they often hold about the efficacy of pain treatments, have also been argued to represent a major challenge for pain professionals (Strous et al., 2006). This adds up to the potential stress of care in this population. In fact, there is recent evidence to suggest that physicians working in pain clinics show particularly high levels of burnout compared with other specialties, which are characterized by high emotional exhaustion and depersonalization, together with reduced personal accomplishment (Riquelme et al., 2018). Also importantly, the repeated exposure of the healthcare professionals to the pain of others has been argued to decrease their empathy (an important component of burnout), ultimately leading to underestimate the patient’s pain (Grégoire et al., 2016), which is likely to have negative consequences on patient care.

As noted in recent systematic reviews about mindfulness programs for healthcare professionals, these programs are potentially useful for this population (Burton et al., 2017; Lomas et al., 2019; Fendel et al., 2021). However, these investigations also reveal that the number of published studies on the application of mindfulness programs for healthcare professionals is still scarce, especially in a particularly stressed population like healthcare professionals working in pain clinic. Also importantly, review studies have shown that most research on the topic has focused on the effectiveness of mindfulness programs, while research has generally ignored feasibility components (e.g., reach, adherence, and acceptability), which are crucial if a program is to be implemented in daily practice (King et al., 2019). Consequently, the present study will assess the feasibility of an 8-week mindfulness-based program to reduce the burnout levels of the staff of a pain clinic in Spain. We expect that the training will be feasible (i.e., a large percentage of potential participants will join the program, they will actively participate in it, and will be satisfied with the program). We also expect that preliminary analyses of effectiveness will show a reduction of burnout and increases mindfulness skills, self-compassion, and empathy levels.

## Materials and methods

A longitudinal study with a within subject pre/post design was conducted in a tertiary hospital pain clinic between April and May 2018.

The pain clinic is categorized as a third level health institution according to the standards and quality recommendations of the Spanish Government. Its area of influence covers a population of almost 450.000 residents and it is a center of reference for the assessment and treatment of complex patients with chronic pain. The demand for care is therefore very high and involves 1,450 new medical appointments, 4,500 follow-up consultations, and 4,000 interventional procedures yearly. The sample included the whole staff, which comprises 6 anesthesiologists, 2 nurse practitioners, 1 nursing assistant, and 1 administrative staff. Except for a few anesthesiologists who develop part of their activities outside the clinic (operating theatres, preoperative consultation, or medical wards), the rest of the workforce (55%) have exclusive dedication to the pain clinic.

### Measures

As recommended in recent guidelines (Pearson et al., 2020), feasibility calculations will include several areas of research, such as reach (i.e., the percent of individuals willing to participate, which will be calculated as program participation rates), adherence (i.e., the degree to which the program is implemented as expected in terms of content or frequency, which will be assessed in the present study as the frequency of participant involvement in the virtual group during the program), acceptability (i.e., the perception that the program is agreeable, which will be evaluated with a measure of satisfaction with the program), and calculations of preliminary effectiveness (with a special emphasis on burnout, in line with the literature overview presented at the beginning of the work).

To evaluate the effectiveness of the program, the same assessment protocol was administered twice at the beginning and the end of the program. This consisted of the following questionnaires:

- The Maslach Burnout Inventory – Human Service Survey (Seisdedos, 1997) was administered to evaluate three components of burnout:
o Emotional exhaustion addresses feelings of being emotionally overwhelmed and exhausted by work (e.g., “I feel emotionally drained from my work”). Higher scores correspond to greater experienced burnout, with scores ranging from 0 to 54.
o Depersonalization measures a tendency to be unfeeling and to have an impersonal response toward recipients of one’s service, care treatment, or instruction (e.g., “I feel I treat some patients as if they were impersonal objects”). Higher scores correspond to greater experienced burnout, with scores ranging from 0 to 30.
o Personal accomplishment evaluates feelings of competence and successful achievement in one’s work (e.g., “I deal very effectively with the problems of my patients”). Lower scores correspond to greater experienced burnout, with scores ranging from 0 to 48.

Response scales in the Maslach Burnout Inventory consist of a 7-point Likert scale ranging from 0 = “never” to 6 = “every day.”

Each scale measures its own unique dimension of burnout and they should not be combined to form a single burnout scale. Burnout syndrome usually presents with a high score in emotional exhaustion and depersonalization and a low score in personal accomplishment.

- Mindfulness abilities were measured by means of the Five Facets of Mindfulness Questionnaire (FFMQ; Cebolla et al., 2012). The scale differentiates five dimensions of mindfulness, namely:

o Observing: ability to observe and attend one’s inner and outer experiences, such as thoughts, emotions, or sensations (e.g., “I pay attention to sensations, such as the wind in my hair or sun on my face”).
o Describing: ability to represent or give an account of experiences in words (e.g., “Even when I’m feeling terribly upset, I can find a way to put it into words”).
o Acting with awareness: ability to engage fully in activities in the present moment (e.g., “I rush through activities without being really attentive to them”).
o Non-judging of inner experience: ability to adopt a non-evaluative position towards experiences (e.g., “I tell myself that I should not be thinking the way I’m thinking”).
o Non-reactivity to inner experience: ability to let the feelings flow avoiding overreaction or refusal (e.g., “In difficult situations, I can pause without immediately reacting”).

Response scales in the FFMQ use a 5-point Likert scale ranging from 1 = “never” to 5 = “very often or always.” Scores in the subscales range from 8 to 40 except for “non-reactivity to inner experience,” where scores have a 7–35 range.

Scores in the global FFMQ range from 39 to 195. Higher scores should be interpreted as representing greater awareness or mindfulness.

- The Jefferson Scale of Empathy (Alcorta-Garza et al., 2016) was used to measure the ability to understand the other person’s pain, suffering, and perspective, combined with an ability to communicate this understanding and an intention to help (e.g., “Empathy is a therapeutic skill without which a healthcare providers’ success is limited”). Response scales in the questionnaire consist of a 7-point Likert scale ranging from 1 = “totally disagree” to 7 = “totally agree.” Scores in the scale can range from 20 to 140, with higher scores corresponding to greater empathy.
- A 12-item, short version of the Self-Compassion Scale (SCS) was administered to evaluate the ability to show self-compassion without judging (Garcia-Campayo et al., 2014). Three subscales can be obtained from the questionnaire:

o Self-kindness evaluates the tendency to be warm and understanding toward ourselves when we suffer, fail, or feel inadequate (e.g., “I’m kind to myself when I’m experiencing suffering”).
o Common humanity is a component of self-compassion that involves recognizing that suffering and personal inadequacy are part of the shared human experience (e.g., “When I feel inadequate in some way, I try to remind myself that feelings of inadequacy are shared by most people”).
o Mindfulness evaluates the ability to take a balanced approach to our negative emotions so that feelings are neither suppressed nor exaggerated (e.g., “When something painful happens, I try to make a balanced view of the situation”).

Response scales in the SCS use a 5-point Likert scale with labels 1 = “almost never” to 7 = “almost always.” Scores in the scale can range from 12 to 60, with higher scores reflecting greater self-compassion. Both the subscales and a total self-compassion score have been used in past research (Garcia-Campayo et al., 2014).

Other variables collected in the study were the following:

- Socio-demographic data. This included age (age 35 or under/36-50/over 50), sex, marital status (single/in a relationship), and occupation (doctor/nurse/assistant nurse/administrative staff).
- Previous experience in mindfulness/meditation (1 = “yes”/0 = “no”).

### Procedure

The first phase of the study consisted of an introductory session, which was conducted by one of the present study authors, who has been accredited with a master’s degree in mindfulness (University of Barcelona). Basic concepts and fundamentals, as well as a short-guided practice, were provided. The purpose of this session was both to disseminate information and to inspire and raise awareness about mindfulness practices amongst the participants.

All staff members were then offered the opportunity to participate in an 8-week program that consisted in guided practical sessions held in the pain clinic before the start of the working day. All the staff voluntarily agreed to participate.

Right after the introductory session, the previously described questionnaires (pre-program) were completed. Next, a virtual group was created using a social media platform on the smartphone (i.e., WhatsApp). This virtual group using technology, which is a novel practice compared to past research (Lomas et al., 2019; Fendel et al., 2021), allowed full access to all the psychoeducational content and audiovisual material daily used during practices. This allowed great flexibility in repeating sessions individually according to one’s availability. All the participants agreed to participate in this virtual group. Examples of the digital content used can be found in Appendix I.

The program consisted of daily face-to-face 10-min sessions for 8 weeks.

At the beginning of each week, early on Monday morning, the participants were sent a pre-session text with information on their smartphones so that they could prepare for the week’s topic. In the group sessions, depending on the weekly topic, the study coordinator selected audiovisual material from specialized web sites lasting between 5 and 8 min. These materials changed daily and were also sent to the participants *via* the virtual group after each session. The reason for doing this was to offer the participants different practical tools (varying tones and cadences of voice, silences or ambient sounds, *etcetera*) to be used for practice.

**Figure 1:**
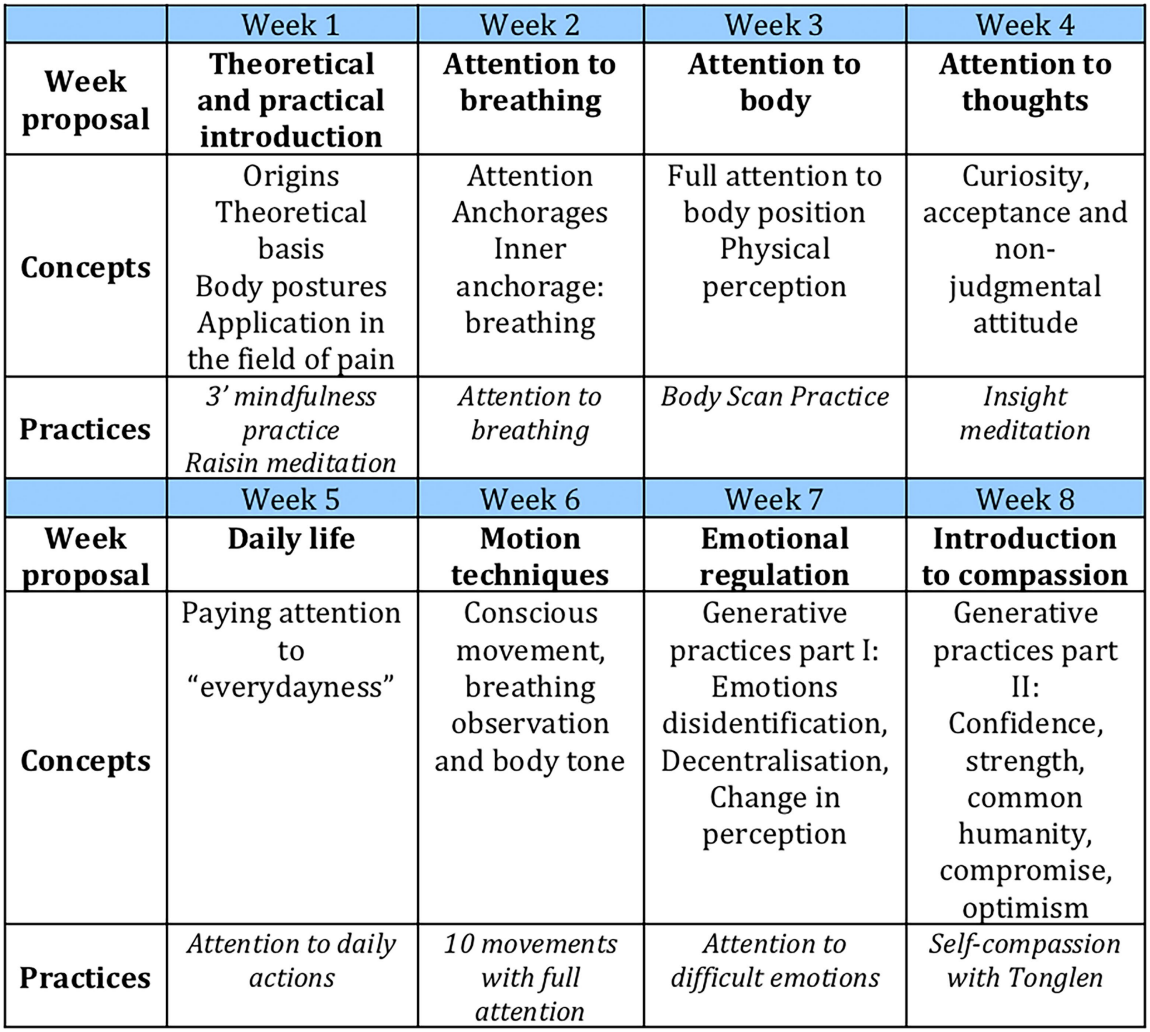
Contents of the training programme.

Session topics were based on the MBSR program designed by Jon Kabat-Zinn and used at the Medical Center of the University of Massachusetts (Kabat-Zinn, 1982) and included: origins and theoretical basis, attention to breathing, attention to body position, attention to thoughts, daily life activities, and conscious movements. The last two weeks were focused on generative practices (emotional regulation and introduction to compassion). The concepts and practices addressed are detailed in Figure 1.

Although the program was conducted in a hospital environment with an intense health care activity, the aim of the study was to conduct group on-site sessions. This condition was possible thanks to the specific features of the pain clinic and the similarity of the working hours between most professionals. The participants arrived an hour earlier to work to avoid disrupting the flow of activity. During the weekends, the practice was carried out individually, based on personal circumstances.

The program took place in a meeting room at the pain clinic. The room was selected to provide a relaxed, low sensory environment that would facilitate a warm and intimate atmosphere. The sessions began with the use of a singing bowl because of its ability to produce harmonic sounds that lead to serenity, mental balance, and reduced feelings of tension, which facilitates a meditative state (Goldsby et al., 2017). Except for the week when the body motion practices were performed, the rest of the sessions were carried out with the participants seated around a round meeting table.

The participants received at least one face-to-face session from Monday to Friday and were encouraged to do extra practicing using the audiovisual material that best suited their needs from the repertoire shared in the social media group. Frequent reminders were sent *via* smartphone to promote practice based on the week session. At the end of the program, the participants completed the assessment protocol again (post-program).

### Data analysis

Description of the sample was performed using demographic and professional categorical and quantitative variables. The analyses included frequency and proportion for categorical variables, and median and inter-quartile range for quantitative variables due to the reduced sample size. The values of these variables and the results of the questionnaires were both as a whole and, in an exploratory manner, segmented by professional categories (physicians vs. non-physicians). This was done because the burnout associated with care provision was expected to be different across employees, as reported in previous research (Chou et al., 2014).

Given the sample size, preliminary calculations of effectiveness were made using a Mann–Whitney U test for continuous variables. Wilcoxon signed-rank test was used to compare pre/post-program results. Statistically significant differences were considered at *p* < 0.05. Wilcoxon effect sizes will be provided. Interpretations tend to be: 0.10–0.3 (small effect), 0.30–0.5 (moderate effect), and ≥ 0.5 (large effect; Tomczak and Tomczak, 2014). Due to the small sample size and the uncontrolled design of the study, the results of the statistical tests should be interpreted with caution.

Statistical analyses were performed using the IBM SPSS Statistics program v. 22. (IBM Corp, 2013).

## Results

Socio-demographic and professional characteristics of the 10 participants are described in Table 1. Most of them were physicians (60%), women (80%), and over 35 years of age (90%). None of them had previous experience with mindfulness practice.

**Table 1:** Sociodemographic and professional characteristics.

N° participants		10
Age	Mean (SD)	46.5 (8.91)
	<= 35 years	1 (10%)
	36–50 years	5 (50%)
	>50 years	4 (40%)
Gender	Female	8 (80%)
Professional category	Medical doctor	6 (60%)
	Nurse	2 (20%)
	Assistant nurse	1 (10%)
	Administrative staff	1 (10%)
Marital status	In a relationship	8 (80%)
N° of children	None	4 (40%)
	1 child	4 (40%)
	2 children	2 (20%)

All values reflect frequencies and percentages, except for the first line in age.

Overall, the results suggested good feasibility of the program. Regarding reach, which was calculated as the healthcare professionals’ participation in the daily sessions from Monday to Friday, 90% of the potential participants (9 out of 10) agreed to participate in the program. Only one physician reported being too busy to participate. Taking adherence, the analysis of the interaction in the virtual group indicated that there was a high participation rate during the program (95% of the participants accessed the platform daily from Monday to Friday to check the messages promoting practice).

Finally, at the end of every practice and at the end of the study, participants were asked to rate each session informally on a scale ranging from “0 = completely dissatisfied” to “5 = completely satisfied” to evaluate satisfaction with the program components. It became evident that the last two components, that is, generative practices, proved less comfortable. The remaining components were rated by all the participants as either completely satisfactory or mostly satisfactory. They were also asked to rate whether the group format suited their needs, which also received high satisfaction rates (from 4 to 5 on the aforementioned Likert-scale response rate from 0 to 5).

Regarding preliminary effectiveness, results of the pre/post-program measures for the whole sample are shown in Table 2. Burnout scores remained unaltered across the whole sample (all *p* > 0.05). The same finding applied to empathy. Changes at the end of the program, however, were revealed for the secondary outcomes of mindfulness and self-compassion components (all *p* < 0.05). To explore whether burnout, but also mindfulness and related variables, differed across professionals (physicians vs. non-physicians), baseline scores were compared (Table 3). The analyses only showed a trend for burnout (emotional exhaustion), in the direction of physicians being more exhausted [24.5(8.25–28.5) vs. 7(3.5–9.75); *p* = 0.069]. Medical professionals showed lesser non-reactivity [14(17.5–18.5) vs. 21.5 (19.5–26.75); *p* = 0.024] and common humanity [5.5 (4.25–5.75) vs. 6.75(5.75–8.13); *p* = 0.047].

**Table 2:** Results of pre/post-intervention questionnaires.

Results	Pre-intervention	Post-intervention	*p*	Z	*r*
*Burnout*					
Emotional exhaustion	9.5 (5.8–26.5)	9.5 (4.8–16.5)	0.110	−1.599	0.53
Depersonalization	5.5 (0.75–8.5)	4 (2.75–6)	0.590	−0.538	0.18
Personal accomplishment	40 (36.25–44.25)	42 (38–47)	0.550	−0.598	0.20
*Mindfulness*					
Global	106.5 (97.25–117.25)	116 (109.5–122)	0.037	−2.090	0.70
Observation	21 (16–25.25)	32 (28.75–34)	0.007	−2.703	0.90
Description	24 (23.3–24.25)	26.5 (24–27.25)	0.012	−2.512	0.84
Acting with awareness	18 (15–31.75)	18 (11.75–2.5)	0.038	−2.077	0.69
Non-judging	22 (19.25–25.25)	17.5 (14.25–23)	0.038	−2.077	0.69
Non-reactivity	18.5 (16.75–2.75)	22 (2.75–23.5)	0.028	−2.201	0.73
Empathy	82 (77.75–92.5)	82.5 (8.75–84.5)	0.798	−0.256	0.09
*Self-compassion*					
Global	37.5 (35.75–38.5)	37 (33.75–4.5)	0.678	−0.416	0.14
Self-kindness	6 (4.75–7.5)	7.8 (6–8.63)	0.012	−2.501	0.83
Common humanity	5.5 (5.25–6.6)	7 (5.9–8.5)	0.011	−2.536	0.85
Mindfulness	5.5 (4.25–8.25)	7 (6.5–8.75)	0.009	−2.611	0.87

Data represented as median (inter-quartile range). “Z” corresponds to Wilcoxon statistic; “r” corresponds to Wilcoxon effect size, where interpretations tend to be: 0.10–0.3 (small effect), 0.30–0.5 (moderate effect), and ≥0.5 (large effect).

**Table 3:** Results of baseline questionnaires segmented by professional category.

	Physicians	Non-physicians	*p*	Z	*r*
*N*	6 (60%)	4 (40%)			
*Burnout*					
Emotional exhaustion	24.5 (8.25–28.5)	7 (3.5–9.75)	0.069	−1.818	0.61
Depersonalization	7 (3–1.75)	3 (0.25–7.75)	0.198	−1.287	0.43
Personal accomplishment	41.5 (36.75–45)	39.5 (32.75–43.25)	0.451	−0.753	0.25
*Mindfulness*					
Global	108.5 (96.25–119.25)	102.5 (96–117.25)	0.670	−0.426	0.14
Observation	18.5 (14.75–21.5)	25.5 (21.25–31.25)	0.054	−1.913	0.64
Description	24 (23.25–25)	24 (21–24)	0.278	−1.287	0.43
Acting with awareness	28 (15–34)	16.5 (13–18.5)	0.198	−0.753	0.25
Non-judging	23.5 (2.75–29.75)	19 (11–23.25)	0.133	−1.931	0.64
Non-reactivity	14 (17.5–18.5)	21.5 (19.5–26.75)	0.024	−1.0	0.33
Empathy	79.5 (73.25–84.25)	89.5 (82–99.25)	0.052	−1.942	0.65
*Self-compassion*					
Global	38 (35.75–38.5)	36.5 (32.25–39.25)	0.515	−0.652	0.22
Self-kindness	5.5 (3.75–7.25)	6.5 (6–8.5)	0.281	−1.079	0.36
Common humanity	5.5 (4.25–5.75)	6.75 (5.75–8.13)	0.047	−1.986	0.66
Mindfulness	5 (3.13–8.25)	6.5 (5.125–9)	0.336	−0.962	0.32

Data represented as median (inter-quartile range). “Z” corresponds to Wilcoxon. “r” corresponds to Wilcoxon effect size, where interpretations tend to be: 0.10–0.3 (small effect), 0.30–0.5 (moderate effect), and ≥ 0.5 (large effect).

Our next and final step included an exploratory analysis of the differential program effect across professionals (Table 4). Significant reductions in burnout, namely emotional exhaustion (which was slightly higher in physicians), were only revealed in physicians [24.5 (8.25–28.5) vs. 11 (4.75–18.75); *p* = 0.028]. Similarly, changes in mindfulness and self-compassion were only reported by physicians. Specifically, an increase in observation [18.5(14.75–21.5) vs. 30.5(28–34); *p* = 0.028], non-reactivity [17.5(14–18.5) vs. 21.5(20.75–22.25); *p* = 0.028], common humanity [5.5(3.75–7.25) vs. 7.5(5.88–8.25); *p* = 0.043], and mindfulness [5(3.13–8.25) vs. 6.75(5.88–8.5); *p* = 0.046] was revealed. On the contrary, a reduction in acting with awareness [28(15–34) vs. 18(11.25–22); *p* = 0.028] and non-judging was observed [23.5(20.75–29.75) vs. 17(14.25–23.5); *p* = 0.028]. No changes were observed in the sample of non-physicians.

**Table 4:** Results of pre/post-intervention questionnaires segmented by professional category.

N° Subjects	Physicians					Non-physicians				
	6 (60%)					4 (40%)				
Questionnaires	Pre-	Post-	*p*	*Z*	*r*	Pre-	Post-	*p*	*Z*	*r*
*Burnout*										
Emotional exhaustion	24.5 (8.25–28.5)	11 (4.75–18.75)	0.028	−2.201	0.73	7 (3.5–9.75)	9 (4.25–13.75)	0.109	−1.604	0.73
Depersonalization	7 (3–1.75)	4.5 (3–6)	0.225	−1.214	0.40	3 (0.25–5.75)	2 (2.25–9)	0.257	−1.134	0.40
Personal accomplishment	41.5 (36.75–45)	45 (41–47)	0.084	−1.725	0.58	39.5 (32.75–43.25)	28 (26.75–42.2)	0.285	−1.069	0.58
*Mindfulness*										
Global	108.5 (96.25–119.25)	116.5 (104.75–122)	0.249	−1.153	0.38	102.5 (96–117.25)	116 (118.5–127.75)	0.068	−1.826	0.38
Observation	18.5 (14.75–21.5)	3.5 (28–34)	0.028	−2.201	0.73	25.5 (21.25–31.25)	33 (29.75–34)	0.141	−1.473	0.73
Description	24 (23.25–25)	26.5 (23–27.25)	0.098	−1.656	0.55	24 (21–24)	26.5 (24.5–28.5)	0.068	−1.826	0.55
Acting with awareness	28 (15–34)	18 (11.25–22)	0.028	−2.201	0.73	16.5 (13–18.5)	18 (12.25–20)	0.414	−0.816	0.73
Non-judging	23.5 (2.75–29.75)	17 (14.25–23.5)	0.028	−2.201	0.73	19 (11–23.25)	20 (11.25–22.75)	0.785	−0.272	0.73
Non-reactivity	17.5 (14–18.5)	21.5 (2.75–22.25)	0.028	−2.201	0.73	21.5 (19.25–26.75)	23.5 (19.75–26.5)	1	<0.001	0.73
Empathy	79.5 (73.25–84.25)	82 (79.5–84.75)	0.527	−0.632	0.21	89.5 (82–99.25)	83.5 (81.5–85.5)	0.273	−1.095	0.21
*Self-compassion*										
Global	38 (35.75–38.5)	38 (36.5–4.5)	0.414	−0.816	0.27	36.5 (32.25–39.25)	33.5 (3.75–4.75)	0.854	−0.184	0.27
Self-kindness	5.5 (3.75–7.25)	7.5 (5.88–8.25)	0.058	−1.897	0.63	6.5 (6–8.5)	8.25 (6.5–9.63)	0.109	−1.604	0.63
Common humanity	5.5 (4.25–5.75)	6.5 (5.88–8.5)	0.043	−2.023	0.67	6.75 (5.75–8.13)	7.75 (5.875–8.88)	0.102	−1.633	0.67
Mindfulness	5 (3.13–8.25)	6.75 (6.13–8.75)	0.046	−1.997	0.67	6.5 (5.13–9)	7.5 (6.63–9.5)	0.066	−1.841	0.67

Data represented as median (inter-quartile range). “Z” corresponds to Wilcoxon statistic; “r” corresponds to Wilcoxon effect size, where interpretations tend to be: 0.10–0.3 (small effect), 0.30–0.5 (moderate effect), and ≥0.5 (large effect).

## Discussion

Over a decade ago some authors started advocating that special attention should be paid to health care providers (Butterfield, 1988) and this remains relevant to the present date (Martín-Brufau et al., 2020; Liu et al., 2021). Despite this call for change, dissatisfaction and distress have continued to increase. Given the inherent difficulties to influence personally in those variables related to work overload or administrative burden, this study aimed to implement and explore the feasibility of a mindfulness training program for healthcare professionals in a high complexity pain clinic. To the best of our knowledge, this is the very first study of this type in this population (e.g., healthcare professionals working at a pain clinic) and using technology to facilitate, support, and encourage daily practice, which opens avenues for new research and might help inspire future investigations and expands past similar research (Lebares et al., 2018; Fendel et al., 2020). Even though the reduced sample size limits the generalizability and robustness of findings, our study suggests that the implementation of a MBSR in real-life settings in a pain clinic might be feasible (e.g., it can be implemented with high satisfaction and participation rates and leads to an acceptable interference on the daily routine of care). We also obtained preliminary confirmatory evidence that the program might promote mindfulness and self-compassion skills and exploratory evidence on benefits for burnout, but only in physicians.

Regarding feasibility, reach, adherence, and acceptability results were excellent. It is important for implementation purposes that almost all the healthcare professionals at the Pain Clinic were willing to participate in the program and those who participated were highly adherent and manifested a high degree of satisfaction with the study design and found the format suitable for their needs. They also expressed an eager willingness to repeat the experience. However, not all the participants reported the same satisfaction with all the practices conducted. This variability relied both on the week proposal and the nature of the audiovisual material. It was apparent that participants reported more discomfort with the last sessions of the program (difficult emotions and self-compassion), as evidenced in previous studies (Gracia Gozalo et al., 2018). These components are fundamental in mindfulness programs, so their exclusion does not seem appropriate. However, strategies like addressing the content differently (e.g., more progressively or including extra sessions to facilitate practice) could be considered in future research.

Regarding the secondary outcomes of the study, which are related to the effectiveness of the program, the study evidenced group changes in self-compassion and mindfulness skills, but not in empathy and burnout. Regarding empathy, the result might be attributable to a ceiling effect, which has been also reported in past research with health care professionals (Boellinghaus et al., 2014). While it is possible that this was not as specifically addressed as mindfulness components in the program, it is also possible that baseline empathy (which was high) might explain this. These results are consistent with those obtained in earlier studies (Amutio Kareaga et al., 2008; Gracia Gozalo et al., 2018), but at this stage it is impossible to conclude whether the program was ineffective or if baseline scores were already high in this construct and a ceiling effect was responsible for the lack of change.

An interesting finding was obtained for burnout in our exploratory analyses. In particular, our study revealed that a brief mindfulness program reduced burnout, particularly emotional exhaustion, in physicians working at our pain clinic. Even though this change did not occur in non-physicians, this might be attributable to the lower levels of burnout in this population, as indicated in our results. Therefore, our findings are in line with research suggesting that physicians are a population at particular risk for burnout (Grover et al., 2018). Also consistent with past research, the fact that changes only occurred in the subgroup with higher burnout might be interpreted as showing that there might be more room for improvement in persons with higher suffering (Osma et al., 2021). This might be important for implementation purposes and personalization of programs to the specific needs of individuals, as in stepped-care models where more intensive programs are offered to individuals according to their baseline severity (Bailey et al., 2022).

In the case of pain clinics, it must be emphasized that pain is a complex personal experience and, especially in chronic pain, it is deeply influenced by psychological mechanisms. There is a shortage of psychology experts in our pain clinic, entailing that non-expert professionals must deal with patients’ affective components. Without the adequate resources to cope with these challenges, this could also play a role in burnout and needs to be addressed. In fact, as noted earlier, in our study burnout in the physicians was slightly higher than in the rest of the staff, which would be in line with the previous. The positive benefits of this mindfulness program on the pain clinic’s physicians’ burnout extends previous evidence on the effectiveness of mindfulness-based programs on other health care providers (Morgan et al., 2015; Gracia Gozalo et al., 2018). The effect sizes found in the present study, which generally ranged from moderate to large, are also consistent with past similar research as noted in recent reviews (Burton et al., 2017; Lomas et al., 2019). A recent review, however, indicated only small effect sizes for burnout in physicians (Fendel et al., 2021). While the present study results should be taken with caution due to the reduced sample size, it is possible that the social media component included in the present study helped incorporate some of the concepts more successfully, which might explain the good results obtained for burnout levels in physicians.

The stress inherent to health care is known to have a negative impact on health care providers, both personally and professionally (Shanafelt et al., 2002). Considering the deleterious effects of a health care system weakened by stress and burnout, it is imperative to focus on promoting good mental health amongst professionals. Moreover, stress and burnout are associated with low level of satisfaction from patients (Garman et al., 2002), which might lead to a vicious cycle of patient dissatisfaction-physician burnout. Provision of resources to the professionals to manage levels of stress, feelings, attention span or empathy becomes a necessity to provide a comprehensive care of this complex kind of patients. Therefore, it appears obvious to us that strategies directed at preventing burnout development, lack of empathy, or self-compassion are needed. Although this study is done prior to the covid-19 pandemic, its results could support the use of this strategy at a time when burnout is so widespread in health care personnel globally (Gualano et al., 2021) and psychological support for this population is more necessary than ever (Önen Sertöz et al., 2021).

The methodological limitations of the study include a reduced sample size and the absence of a control group. It should also be noted that participants may present a positive bias towards the program, as they all accepted to participate in the study because they were interested in mindfulness. While acknowledging this, it is important to note that none of them had previous experience with mindfulness or meditation, which could have represented an additional bias in the sample. A final concern is the inability to monitor everyone’s practice between sessions. With the availability of smartphones, future studies could evaluate daily practice using an app for ecological momentary assessment (Colombo et al., 2020).

In conclusion, the 8-week mindfulness-based program described in the present investigation might be a feasible and potentially effective method that can be easily implemented to promote mindfulness and self-compassion in healthcare professionals of specialized pain clinics. Also importantly, potential benefits in burnout were found for physicians. It is obvious that healthcare professionals need support in addressing the wide range of potential stressors inherent to their work environment. Efforts to reduce burnout and promote satisfaction in healthcare professionals should be encouraged and we believe this kind of mindfulness strategies may lead to healthier behaviors in our professionals and, therefore, to a better patient care.

## Data availability statement

The raw data supporting the conclusions of this article will be made available by the authors, without undue reservation.

## Ethics statement

The studies involving human participants were reviewed and approved by Comité de Ética de Investigación con Medicamentos y Comisión de Proyectos de Investigación del Hospital Universitari Vall D'Hebron (reference PR(ATR)441/2018). The patients/participants provided their written informed consent to participate in this study.

## Author contributions

AS designed the study, conducted the study, collected the data, analyzed the data, and prepared the manuscript. CS-R designed the study, analyzed the data, and prepared the manuscript. MP-C collected the data and analyzed the data. JM and ÁM conducted the study and prepared the manuscript. AA and RG designed the study and prepared the manuscript. All authors have read and approved the final version of the manuscript.

## Funding

This research was funded by Universitat Jaume I, grant number UJI-A2020-03.

## Conflict of interest

The authors declare that the research was conducted in the absence of any commercial or financial relationships that could be construed as a potential conflict of interest.

## Publisher’s note

All claims expressed in this article are solely those of the authors and do not necessarily represent those of their affiliated organizations, or those of the publisher, the editors and the reviewers. Any product that may be evaluated in this article, or claim that may be made by its manufacturer, is not guaranteed or endorsed by the publisher.
