# Supplementary material for: Feasibility of a brief mindfulness-based program for burnout in pain healthcare professionals
Source: Front Psychol. 2022 Nov 7;13:1009266. doi: 10.3389/fpsyg.2022.1009266 (PMC9677093; doi:10.3389/fpsyg.2022.1009266)
Supplement: Supplementary file 1 [file Data_Sheet_1.DOCX]

Appendix I. Digital material used for the program across weeks

| **Week 1**  https://youtu.be/_CZEEYMXr8Q  https://youtu.be/CR31aV92PLk  https://youtu.be/jehJTsQn3Pw | **Week 2**  https://youtu.be/kNl_JnZP9lM  https://youtu.be/3Xcmk5WmsIU  https://youtu.be/dTF6hDlCVB0  https://youtu.be/FZ18M0iXTqM | **Week 3**  https://youtu.be/dsmfIAyiois  https://youtu.be/EEgBcXEoZ3A  https://youtu.be/t2UFYXYQHjA  https://youtu.be/eoiW6emDyUE |
| --- | --- | --- |
|  |  |  |
| **Week 4**  https://youtu.be/rrN4_hInudY  https://youtu.be/XwcIYkV69T8  https://youtu.be/rrN4_hInudY | **Week 5**  https://youtu.be/O9ZkzJH37Ls  https://youtu.be/j4RPFgCCi7U  https://youtu.be/sqBKUFWjgjw | **Week 6**  https://youtu.be/WKV7laXr_mk  https://youtu.be/jRSgKAScBGo  https://youtu.be/tMu3JW_FyjI |
| **Week 7**  https://youtu.be/G780Sa_wGss  https://youtu.be/6kylDOdRAJY  https://youtu.be/QYF00xD3iVY  https://youtu.be/6hTEyWyFLEQ | **Week 8**  https://youtu.be/wp27d55R2pw  https://youtu.be/w3ghmzeeqDI  https://youtu.be/ZmU2iZemwf0 |  |
